# An RNAi screen of Rho signalling networks identifies RhoH as a regulator of Rac1 in prostate cancer cell migration

**DOI:** 10.1186/s12915-018-0489-4

**Published:** 2018-03-06

**Authors:** Virginia Tajadura-Ortega, Ritu Garg, Richard Allen, Claudia Owczarek, Michael D. Bright, Samuel Kean, Aisyah Mohd-Noor, Anita Grigoriadis, Timothy C. Elston, Klaus M. Hahn, Anne J. Ridley

**Affiliations:** 1 Randall Centre of Cell and Molecular Biophysics, King’s College London, New Hunt’s House, Guy’s Campus, London, SE1 1UL UK; 2 School of Cardiovascular Medicine and Sciences, King’s College London, London, SE1 9NH UK; 3 Department of Pharmacology, University of North Carolina at Chapel Hill, Chapel Hill, NC 27599 USA; 4 School of Cancer and Pharmaceutical Sciences, King’s College London, Guy’s Hospital, London, SE1 9RT UK; 5 School of Cellular and Molecular Medicine, University of Bristol, Biomedical Sciences Building, University Walk, Bristol, BS8 1TD UK; 6 Present address: Internal Medicine Research Unit, Pfizer Inc, Cambridge, MA 02139 USA; 7 Present address: Institute for Cancer Research, 15 Cotswold Road, Sutton, SM2 5NG UK

## Abstract

**Background:**

Cell migration is essential for development and tissue repair, but it also contributes to disease. Rho GTPases regulate cell migration, but a comprehensive analysis of how each Rho signalling component affects migration has not been carried out.

**Results:**

Through an RNA interference screen, and using a prostate cancer cell line, we find that approximately 25% of Rho network components alter migration. Some genes enhance migration while others decrease basal and/or hepatocyte growth factor-stimulated migration. Surprisingly, we identify RhoH as a screen hit. RhoH expression is normally restricted to haematopoietic cells, but we find it is expressed in multiple epithelial cancer cell lines. High RhoH expression in samples from prostate cancer patients correlates with earlier relapse. RhoH depletion reduces cell speed and persistence and decreases migratory polarity. Rac1 activity normally localizes to the front of migrating cells at areas of dynamic membrane movement, but in RhoH-depleted cells active Rac1 is localised around the whole cell periphery and associated with membrane regions that are not extending or retracting. RhoH interacts with Rac1 and with several p21-activated kinases (PAKs), which are Rac effectors. Similar to RhoH depletion, PAK2 depletion increases cell spread area and reduces cell migration. In addition, RhoH depletion reduces lamellipodium extension induced by PAK2 overexpression.

**Conclusions:**

We describe a novel role for RhoH in prostate cancer cell migration. We propose that RhoH promotes cell migration by coupling Rac1 activity and PAK2 to membrane protrusion. Our results also suggest that RhoH expression levels correlate with prostate cancer progression.

**Electronic supplementary material:**

The online version of this article (10.1186/s12915-018-0489-4) contains supplementary material, which is available to authorized users.

## Background

Cell migration is essential for the development of multicellular animals, as well as for immune responses and wound healing. Cell migration also contributes to the development of several human diseases, including cancer, heart disease and chronic inflammatory disorders [1–3]. Many cell-surface receptors and intracellular signalling proteins contribute to cell migration, and inhibitors of some of these molecules are being developed for the treatment of human diseases [4, 5].

Rho GTPases are intracellular coordinators of cell migration signalling. Most Rho GTPases cycle between an active GTP-bound and an inactive GDP-bound form. They are activated by guanine nucleotide exchange factors (GEFs), which stimulate exchange of GDP for GTP, and inactivated by GTPase-activating proteins (GAPs), which stimulate their intrinsic GTPase activity, hydrolysing GTP to GDP [6]. Most Rho family members are post-translationally modified at the C-terminus by addition of lipid groups, which facilitate their interaction with membranes. Some Rho GTPases are regulated by guanine nucleotide dissociation inhibitors (GDIs), which extract them from membranes by binding to the lipid group as well as other regions of the protein [7]. When bound to GTP, Rho GTPases interact with a wide range of downstream effectors to induce signalling, including enzymes, adaptor proteins and regulators of actin polymerisation. Several Rho family members are atypical: They have amino acid substitutions that prevent GTP hydrolysis and are thus constitutively bound to GTP. These include RhoH and Rnd proteins [8, 9]. The activity of these proteins can be regulated by post-translational modifications; for example, Rnd proteins are inhibited by serine/threonine phosphorylation and subsequent binding to 14-3-3 proteins [10], and RhoH can be tyrosine phosphorylated by Src family kinases, which promotes its interaction with ZAP70 in T cells or Syk in mast cells [11–13].

Several groups have used RNA interference (RNAi) screens to identify regulators of cell morphology and/or cell migration. Targeted screens of cytoskeletal regulators as well as whole genome screens have identified new genes involved in cell migration [14–18].

Here we have carried out a focussed RNAi screen to identify which Rho GTPases and their interacting partners regulate cancer cell migration. Unexpectedly, we identified RhoH as a key regulator of cancer cell migration. RhoH is widely assumed to be expressed only in haematopoietic cells based on analysis of mouse tissues [19]. We show here that RhoH is in fact expressed in a wide range of prostate and breast cancer cell lines and human cancers. We demonstrate that RhoH stimulates cell migration by promoting Rac1- and PAK-driven membrane protrusion.

## Results

### An RNAi screen of the Rho network identifies groups of genes with different effects on cancer cell migration

**Fig. 1:**
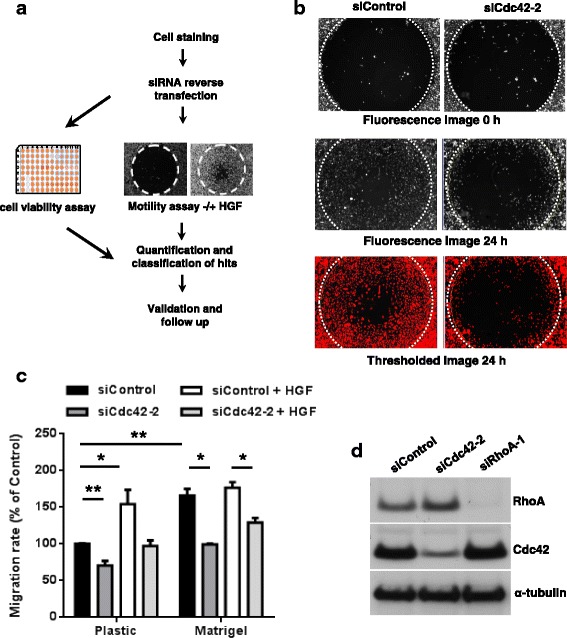
Design and validation of siRNA screen. **a** Schematic representation of the screen design and implementation. PC3 cells were stained with carboxyfluorescein succinimidyl ester (CFSE) and reverse transfected with siRNAs. Cells were then divided and seeded into one 96-well plate to carry out cell viability analysis and two separate 96-well plates to measure migration by Oris™ assay with and without hepatocyte growth factor (*HGF*) (Table 1). Hits from the screen were quantified. **b** Conditions for the migration screen were tested using an siRNA targeting Cdc42 (siCdc42-2, Additional file 1: Table S1) and control siRNA. Images show examples of CFSE-labelled PC3 cells immediately after removal of the Oris™ stopper (0 h) and 24 h later. *Bottom images* are thresholded 24 h images (see Methods). *White dotted circle* indicates wound area at 0 h. **c**. Quantification of the effect of Cdc42 depletion on migration in the Oris™ assay using the screen conditions, comparing cells on uncoated plastic and Matrigel-coated plastic. *n* = 3, mean +/− standard error of the mean (SEM); **p ≤ 0.01, *p ≤ 0.05, Student’s *t* test. **d** Single siRNAs targeting Cdc42 (siCdc42-2), RhoA (siRhoA-1) or control siRNA were tested for knockdown of protein expression using the screen conditions. Cells were lysed 72 h after transfection, and cell lysates western blotted with antibodies to Cdc42 and RhoA, and α-tubulin as a loading control

To investigate which Rho GTPase network components affect cell migration, we used a small interfering RNA (siRNA) library containing pools of four siRNAs targeting each of 202 genes encoding Rho GTPases, RhoGEFs, RhoGAPs and Rho targets, together with four control siRNA pools (Additional file 1: Table S1). The screen method used a modified wound healing assay to study migration (see Methods) with PC3 prostate cancer cells (Fig. 1a). PC3 cells express N-cadherin but not E-cadherin and only make transient cell-cell interactions. They predominantly migrate as single cells and thus resemble cells that have undergone epithelial-to-mesenchymal transition [20, 21].

The assay conditions were established using siRNAs targeting Cdc42 and control siRNA. Migration was evaluated by image analysis comparing the area covered with cells at different times after creation of the ‘wound’. Cells were stained with the fluorescent dye carboxyfluorescein succinimidyl ester (CFSE) to allow rapid quantification of relative cell number in the wound area. Images were treated with a flatten background filter and then thresholded so that differences in CFSE uptake between individual cells did not affect the results (Fig. 1b, see Methods). After 24 h, control siRNA-transfected cells covered approximately 50–60% of the wound area (Fig. 1b). This was established as the optimal time point for the screening, because it allowed the identification of genes that inhibited and enhanced migration.

Cdc42 depletion strongly reduced cell migration (Fig. 1b, c). We compared migration on uncoated plastic with that on Matrigel-coated plastic (Fig. 1c). Although cells migrated faster on Matrigel, the effect of Cdc42 depletion was still clear on uncoated plastic, and this condition was more reproducible for screening than Matrigel coating (unpublished data). Migration was analysed in the presence and absence of hepatocyte growth factor (HGF) (Fig. 1a), which promotes migration and invasion of many cell types [22] and stimulated PC3 cell migration in this assay (Fig. 1c). The siRNA for Cdc42 as well as an siRNA for RhoA showed reliable depletion of proteins under the assay conditions (Fig. 1d).

The siRNA library screen was repeated three times. To identify if any siRNA pools affected cell viability, PC3 cells were plated onto 96-well plates 24 h after transfection, and the fluorescence was measured after 24 and 48 h to monitor cell viability (Fig. 1a). Only a few siRNA pools affected cell number in each round of the screen. Those wells that had significantly fewer cells than the mean in an individual screen and/or did not form a defined wound area were excluded from the migration analysis. Only PLD5, CYBB, PLXNB1 and PLXNB2 siRNA pools affected viability in all three independent screens (Additional file 1: Table S1; Z-score < −1.0).

**Fig. 2:**
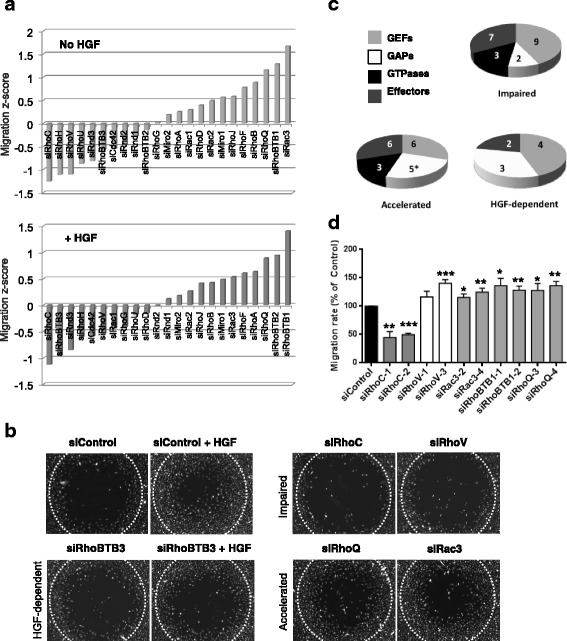
Screen of Rho RNAi library identifies four cell migration phenotypes. The migration screen was performed with PC3 cells transfected with siRNA pools or single siRNAs targeting the indicated genes. After 24 h, cells were seeded at confluence into 96-well plates containing Oris™ stoppers. After 24 h, the stoppers were removed and cells were treated with or without 10 ng/ml HGF for 24 h. Images of cells were acquired immediately after removing the stoppers (0 h) and 24 h later. **a** Graph shows migration Z-scores (see Methods) for the GTPases included in the screen in normal medium (*top panel*) or medium containing HGF (*bottom panel*); *n* = 3. **b** Representative images of migration area (24 h after stopper removal) for representative hits from each phenotypic category: no effect on migration (siControl), accelerated migration (RhoQ, Rac3), impaired migration (RhoC, RhoV), impaired migration only in response to HGF (RhoBTB3). *White dotted circle* indicates wound area at 0 h. **c** Pie charts show number of genes giving each phenotype, based on type of gene (Rho GTPase, RhoGEF, RhoGAP, Rho effector). Note that BCR, which contains both a RhoGEF and a RhoGAP domain, was included as a GAP in the pie chart for accelerated migration and indicated with an asterisk. **d** Migration for cells transfected with the indicated single siRNAs is shown as percentage of control siRNA-transfected cells +/− SEM, *n* = 3; *p ≤ 0.05, **p ≤ 0.01, ***p ≤ 0.001, Student’s *t* test

Screen results were analysed by calculating the migration rate for each individual siRNA pool compared to the mean of all the wells in the screen (Fig. 2a). A Z-score of +/−1.0 was used as the cutoff for significance in each screen. Analysis of the results showed that 51 of the 202 siRNA pools tested significantly affected cell migration in the three screens, which included GEFs, GAPs, effectors and Rho GTPases (Table 1; Additional file 1: Table S1). Of these, 21 siRNA pools inhibited basal cell migration, whereas 20 siRNA pools increased basal cell migration (Fig. 2b, c). Ten siRNA pools only affected HGF-stimulated migration but not basal migration (Table 1).

**Table 1:** List of migration hits from siRNA library screen

	Impaired migration			Accelerated migration			HGF-dependent migration		
	Gene name	Accession number	Z-score	Gene name	Accession number	Z-score	Gene name	Accession number	Z-score
GTPases	*RHOC*	NM_175744	−1.2576	*RAC3*	NM_005052	1.6714			
	*RHOH*	NM_004310	−1.1161	*RHOBTB1*	NM_014836	1.2813			
	*RHOV*	NM_133639	−1.1100	*RHOQ*	NM_012249	1.1527			
RhoGAPs	*ARAP1*	NM_015242	−1.3971	*DLC1*	NM_006094	1.2716	*ARAP3* ^b^	NM_022481	1.3769
	*SRGAP3*	NM_014850	−1.3048	*ARAP2*	NM_015230	1.2509	*ARHGAP24* ^b^	NM_031305	1.1005
				*ARHGAP35*	NM_024342	1.1569	*SRGAP1* ^b^	NM_020762	1.0760
				*ARHGAP4*	NM_001666	1.0155			
RhoGEFs	*DOCK5*	NM_024940	−2.3762	*VAV1*	NM_005428	1.6012	*ARHGEF4* ^a^	NM_015320	−1.6365
	*DOCK11*	NM_144658	−1.7138	*ARHGEF10*	NM_014629	1.1390	*ARHGEF19* ^a^	NM_153213	−1.5797
	*DOCK6*	NM_020812	−1.5419	*PLEKHG6*	NM_018173	1.1295	*ARHGEF14* ^a^	NM_024979	−1.2354
	*FGD2*	NM_173558	−1.4725	*ALS2*	NM_020919	1.1294	*FGD4* ^b^	NM_139241	1.1248
	*DOCK3*	NM_004947	−1.3736	*PREX2*	NM_024870	1.0269			
	*ITSN2*	NM_006277	−1.2293	*RASGRF1*	NM_002891	1.0074			
	*SOS1*	NM_005633	−1.1864						
	*DOCK2*	NM_004946	−1.1098						
	*FGD5*	NM_152536	−1.0918						
Effectors	*NOXA1*	NM_006647	−1.4477	*RHPN1*	NM_052924	1.5692	*CIT* ^a^	NM_007174	−1.0435
	*PIK3R2*	NM_005027	−1.3197	*TWF1*	NM_002822	1.3241	*CDC42EP* ^b^	NM_007061	1.4898
	*CDC42EP2*	NM_006779	−1.2882	*PAK4*	NM_005884	1.2268			
	*PKN3*	NM_013355	−1.2045	*PIK3R1*	NM_181504	1.1572			
	*PAK7*	NM_020341	−1.0864	*MLK4*	NM_032435	1.0711			
	*SMURF1*	NM_020429	−1.0663	*PLXNB1*	NM_002673	1.0377			
	*PAK2*	NM_002577	−1.0318						
Other				*BCR*	NM_004327	1.1558	*RHOBTB3* ^a^	NM_014899	−0.9751

Hits are divided into Rho GTPases, RhoGEFs, RhoGAPs and effectors and subdivided into siRNA pools that impair or accelerate PC3 cell migration or affect only HGF-dependent migration; migration Z-score is indicated relative to the mean migration of all genes included in the screen. Accession number: RefSeq accession number is indicated

*HGF* hepatocyte growth factor

^a^HGF-impaired

^b^HGF-accelerated

It is interesting that siRNA pools for 6 of the 20 (30%) Rho GTPase family members altered cell migration (Fig. 2a). All 20 Rho GTPases are expressed in PC3 cells at the messenger RNA (mRNA) level [23]. We previously showed that siRNA pools for 8 of 20 Rho GTPases affect PC3 cell adhesion to endothelial cells [23]. Also included in the library were siRNAs targeting RhoBTB3, Miro1 and Miro2, which are not classified as Rho GTPases [24] (Additional file 1: Table S1). RhoBTB3 siRNAs impaired migration only when cells were stimulated with HGF. RhoBTB3 is associated with the Golgi and regulates protein stability through the proteasome [25, 26].

In total, siRNA pools for 20 RhoGEFs affected cell migration, of the 70 included in the library (29%). All of the 9 RhoGEF siRNA pools that reduced basal cell migration are reported to be GEFs for Rac and/or Cdc42, consistent with roles of Rac and Cdc42 in lamellipodium-driven migration [27]. For RhoGAPs, 10 of 44 (23%) siRNA pools included in the library had a phenotype. Interestingly, a larger number of RhoGAP siRNA pools enhanced cell migration than decreased migration either in the presence or absence of HGF (Table 1).

Of the 57 Rho effectors in our library, 15 are hits (26%) (Table 1). Some of these also regulate Rho GTPases, such as Smurf1, an ubiquitin ligase for multiple proteins including RhoA [28], for which the siRNA pool inhibited migration. None of the three RhoGDIs had a phenotype in PC3 cells.

siRNA pools targeting some highly homologous GEFs and GAPs had opposite effects on migration, indicating that despite their similar domain structure they are likely to have different functions. These include the RhoGEFs FGD2 and FGD4 and the RhoGAPs ARAP1, ARAP2 and ARAP3 (Table 1). Interestingly, ARAP1/2/3 are RhoGAPs with different specificities for RhoA, Cdc42 and Rac1, which also have ArfGAP and Ras-interacting domains, indicating the importance of these proteins in integrating information from different signalling pathways [29].

Since the siRNA screen was carried out with pools of four siRNAs per gene, we investigated whether the phenotypes were reproducible with individual siRNAs. We chose to focus on the six Rho GTPases that scored as hits: RhoC, RhoH and RhoV siRNA pools decreased migration, whereas Rac3, RhoQ and RhoBTB1 pools increased migration (Table 1). Using two different siRNAs from the pool for each gene, we confirmed that RhoC depletion decreased migration, as previously observed [30]. Rac3, RhoQ and RhoBTB1 depletion reproducibly increased migration (Fig. 2d), in line with the results of the screen. Results for RhoH are reported below. Surprisingly, two RhoV siRNAs individually increased migration (Fig. 2d), but another single siRNA (siRhoV-4) reduced migration (data not shown), suggesting that this one might have dominated the pool as an off-target.

### RhoH regulates PC3 cell migration

Of the hits from our screen, we chose to analyse the effect of RhoH on cell migration in more detail, because it was an unexpected hit: RhoH is considered to be expressed only in haematopoietic cells and has previously only been studied in haematopoietic cells [19]. The *RhoH* gene is present in vertebrates but is not found in other clades [24]; thus it is not present and therefore would not be identified in migration/morphology screens in model organisms such as *Drosophila melanogaster* or *Caenorhabditis elegans*.

**Fig. 3:**
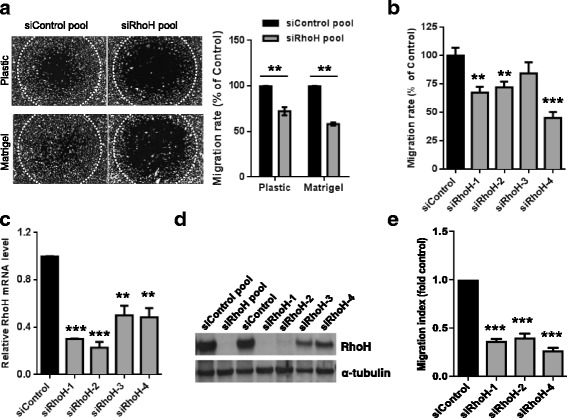
RhoH depletion reduces cell migration. **a** RhoH depletion reduces migration on uncoated plastic or Matrigel-coated plastic. PC3 cells were transfected with a control siRNA pool or the pool of four siRNAs targeting RhoH. After 24 h, cells were seeded around Oris™ stoppers. After 24 h, stoppers were removed and migration was determined 24 h later. *Left*, example images of cells 24 h after removal of stoppers. *White dotted circle* indicates wound area at 0 h. *Right*, graph showing migration as % of siControl +/− SEM, *n* = 3; **p ≤ 0.01, Student’s *t* test. **b** Individual siRNAs targeting RhoH reduce migration. PC3 cells were transfected with indicated siRNAs. Cell migration was determined as in **a**. Graph shows migration as % of siControl +/− SEM, *n* = 3; **p ≤ 0.01, ***p ≤ 0.001, Student’s *t* test. **c** Relative mRNA expression levels following RhoH knockdown, compared to control siRNA-transfected cells. Levels were measured by quantitative real-time PCR (qPCR). *n* = 3; **p ≤ 0.01, ***p ≤ 0.001, Student’s *t* test. **d** Rho siRNAs reduce expression of exogenous RhoH. PC3 cells were transfected with the indicated siRNAs. Cells were transfected with human astrocyte (HA)-RhoH 48 h after siRNA transfection. After 24 h, cell lysates were analysed by western blotting with an anti-RhoH antibody. α-tubulin was used as a loading control. **e** RhoH depletion reduces migration through transwells. PC3 cells were transfected with the indicated siRNAs. Cell migration was determined through transwells with 1% fetal calf serum (FCS) as chemoattractant, and migration index calculated relative to siControl-transfected cells +/− SEM, *n* = 3; ***p ≤ 0.001, Student’s *t* test

To confirm the effect of RhoH depletion on PC3 cell migration, we tested cells transfected with the pool of RhoH siRNA oligos on both uncoated plastic and Matrigel-coated plastic. RhoH depletion reduced cell migration on both substrata in the modified scratch wound assay (Fig. 3a). Three of the four individual siRNA oligos in the pool also reduced migration (Fig. 3b), and all four down-regulated *RhoH* mRNA expression in PC3 cells (Fig. 3c). We were not able to detect endogenous RhoH protein with commercially available RhoH antibodies, although one antibody did detect exogenously expressed RhoH (Fig. 3d). The four individual siRNAs in the RhoH pool all knocked down exogenously expressed RhoH protein.

In addition to the modified scratch wound assay, RhoH depletion reduced PC3 cell chemotaxis through transwells towards a gradient of fetal calf serum (FCS) (Fig. 3e). Together these results indicate that RhoH is required for optimal migration and chemotaxis of PC3 cells.

### RhoH is expressed in a subset of epithelial cancer cell lines and in prostate cancer

**Fig. 4:**
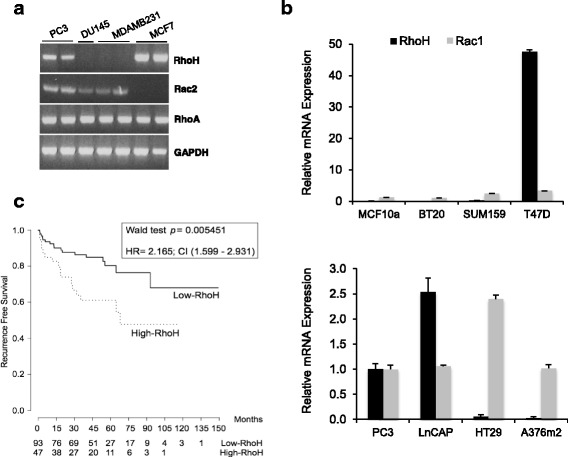
RhoH is expressed in a subset of cancer cell lines and correlates with poor prognosis in prostate cancers. **a**, **b** Several epithelial cancer cell lines express RhoH. **a** RNA was analysed by reverse transcription (RT)-PCR to determine which cell types express mRNA for RhoH, RhoA and Rac2. GAPDH was used as a control. Gel is representative of three experiments. **b** RNA was analysed by qPCR for RhoH and Rac1 expression and normalised to GADPH mRNA. mRNA levels for each cell type are shown relative to levels in PC3 cells. Mean +/− standard deviation for the values of three technical replicates. **c** Expression of RhoH in prostate cancer correlates with poor prognosis. Publicly available RNA expression data from 140 prostate cancers from the Memorial Sloan Kettering Cancer Center (GSE21032) were analysed for RhoH expression. Samples were divided into those with high or low RhoH expression (see Methods). Kaplan-Meier curves show recurrence-free survival (RFS) measured by prostate-specific antigen (PSA) levels, indicating that high RhoH expression correlates with shorter RFS. Numbers under the graph show the number of patients without a recurrence event at a given time point, indicating that high RhoH expression correlates with earlier relapse and shorter time to relapse

Given that *RhoH* is expressed in PC3 cells, we investigated whether other cancer cell lines used in our laboratory also expressed *RhoH*. RNA analysis by polymerase chain reaction (PCR) detected *RhoH* expression in PC3 cells and MCF7 breast cancer cells but not DU145 prostate cancer cells or MDA-MB-231 breast cancer cells (Fig. 4a). All four cell lines expressed *RhoA* and, interestingly, all lines except MCF7 cells expressed *Rac2*, which is also considered to be a haematopoietic cell-specific gene [19].

To find out whether *RhoH* is expressed in other epithelial cancer cell lines, we analysed mRNA expression data in the Cancer Cell Lines Encyclopedia (CCLE) database (http://www.broadinstitute.org/ccle). As expected, the highest *RhoH* expression levels were found in T- and B-cell-derived cancers including lymphoma and leukemia cell lines. *RhoH* was also expressed in a variety of epithelial cancer cell lines, including prostate, breast, colorectal and lung. The 8 prostate cancer cell lines and 58 breast cancer cell lines in the CCLE database expressed *RhoH* mRNA at a wide range of levels. Based on this analysis, we tested for *RhoH* expression in a selection of cancer cell lines, including those with high and low expression based on our CCLE analysis. Quantitative real-time PCR (qPCR) analysis showed that, of the cancer cell lines analysed, *RhoH* mRNA levels were highest in T47D breast cancer cells followed by LNCaP and PC3 prostate cancer cells, whereas *RhoH* mRNA was very low or not detected in a variety of other cancer cell lines (Fig. 4b).

We investigated the relevance of *RhoH* expression in prostate cancer by analysing existing gene expression data associated with clinical parameters of disease progression. Analysis of data from a cohort of patients with prostate cancer from the Memorial Sloan Kettering Cancer Center (MSKCC) [31] showed that high levels of *RhoH* in the tumour samples correlate with decreased recurrence-free survival (RFS) (determined by measuring prostate-specific antigen (PSA) levels) (Fig. 4c). This indicates that increased *RhoH* levels may be associated with poor prognosis in patients with prostate cancer.

### RhoH depletion reduces migration speed and alters migratory behaviour

**Fig. 5:**
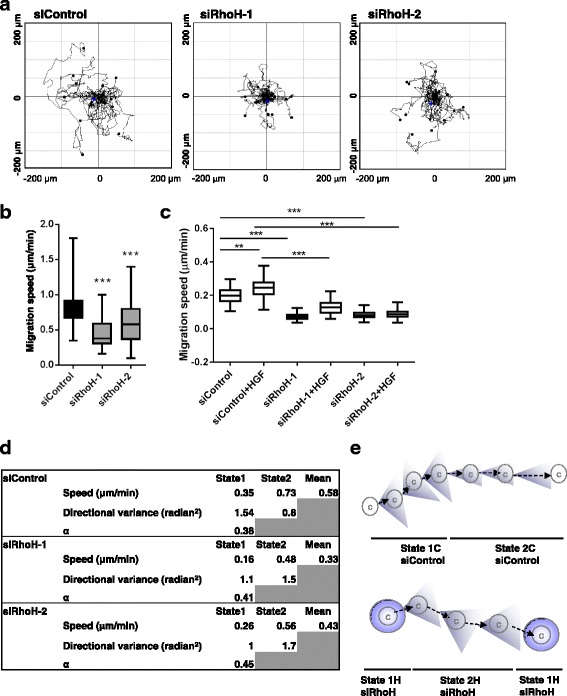
RhoH depletion alters migration behaviour of PC3 cells. RhoH depletion reduces cell migration speed. PC3 cells were transfected with the indicated siRNAs targeting RhoH or control siRNA. Cells were seeded in 24-well dishes 48 h after transfection and monitored by time-lapse microscopy for 24 h. **a** Example cell tracks are shown for each condition, plotted with their starting point at the intersection of the *x*- and *y*-axes. **b** Quantification of PC3 cell migration speed. At least 50 cells were tracked from three independent experiments. *Boxes* of box and whisker plots show median, 25th and 75th percentiles; *whiskers* show minimum and maximum values; ***p ≤ 0.001, Student’s *t* test. **c** Effect of RhoH on HGF-induced migration. 48 h after siRNA transfection, cells were transferred to 0.1% FCS with or without 20 ng/ml HGF. Cells were monitored by time-lapse microscopy for 24 h, with an image taken every 4 min. At least 100 cells were tracked from three independent experiments. *Boxes* of box and whisker plots show median, 25th and 75th percentiles; *whiskers* show minimum and maximum values; **p ≤ 0.01, ***p ≤ 0.001, Student’s *t* test. **d** Data for State 1 and State 2, showing mean migration speed, directional variance (over a time period of 8 min) and proportion of total time spent in State 1 across all cells (α). **e** Analysis of cell tracks indicated two modes of migration, State 1 and State 2, with different migration speeds and persistence (directional variance)


We next investigated whether RhoH depletion affected random migration of subconfluent cells. Two siRNA oligos were chosen for further studies, siRhoH-1 and siRhoH-2, because they gave the strongest knockdown of *RhoH* mRNA and exogenously expressed RhoH protein (Fig. 3c, d). These two siRNAs reduced the random migration speed of single PC3 cells both with and without HGF addition (Fig. 5a–c; Additional file 2: Movie S1, Additional file 3: Movie S2 and Additional file 4: Movie S3).

**Additional file 2: Movie S1.** Random migration of PC3 cells transfected with siControl. One frame every 8 min. Scale bar 20 μm. (AVI 1036 kb)



**Additional file 3: Movie S2.** Random migration of PC3 cells transfected with siRhoH-1. One frame every 8 min. Scale bar 20 μm. (AVI 610 kb)



**Additional file 4: Movie S3.** Random migration of PC3 cells transfected with siRhoH-2. One frame every 8 min. Scale bar 20 μm. (AVI 585 kb)


Detailed analysis of the cell migration tracks (see Methods) showed that control PC3 cells adopted one of two migration states during random migration (Fig. 5d, e). In State 1, the control cells had a slower migration speed and lower directional persistence (as indicated by a higher directional variance), whereas in State 2, cells had a higher migration speed and higher persistence. Following RhoH depletion, we could still identify two migration states, but with distinct properties to those of the control cells: In State 1, cells did not migrate significantly, whereas in State 2, cells had low migration speed and low persistence (high directional variance), similar to State 1 of the control cells (Fig. 5d, e). These results indicate that RhoH is important for both migration speed and directional persistence of cell movement.

### RhoH affects cell shape and actin distribution

**Fig. 6:**
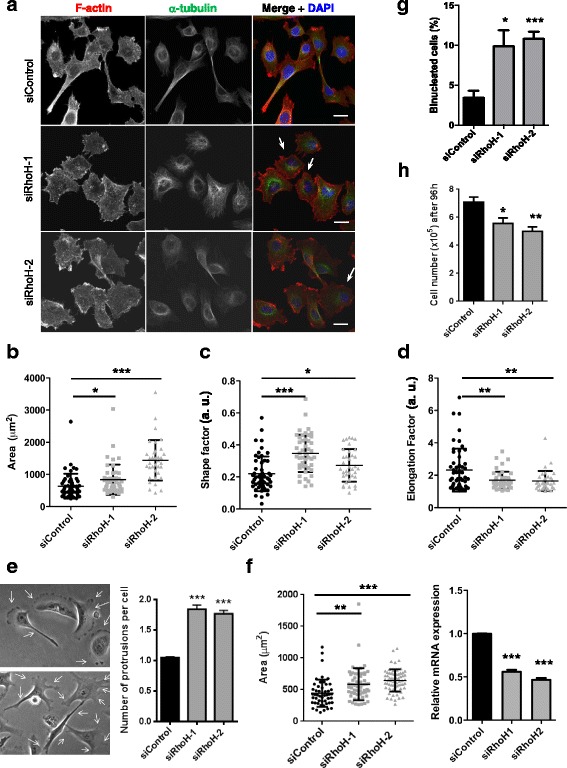
RhoH affects cell shape in PC3 cells. Analysis of cell shape. PC3 cells were transfected with the indicated siRNAs targeting RhoH or control siRNA. After 48 h, cells were seeded on Matrigel-coated glass coverslips. 72 h after transfection, cells were fixed and stained for F-actin (Alexa Fluor 546 phalloidin), tubulin (fluorescein isothiocyanate (FITC)-labelled anti-tubulin antibody) and DNA (4′,6-diamidino-2-phenylindole (DAPI)). **a** Representative images of cells. Scale bar 10 μm. *Arrows* indicate examples of binucleated cells. **b**–**d** PC3 cell area, shape factor and elongation factor were measured using ImageJ from F-actin-stained images. Scatter dot plots show mean +/− SEM; at least 60 cells per condition were analysed from three independent experiments, *p ≤ 0.05, **p ≤ 0.01, ***p ≤ 0.001, Student’s *t* test. **e** Quantification of the number of extended protrusions during cell migration. Panels show representative images of siControl- or siRhoH-transfected cells. *Arrows* indicate protrusions on migrating cells. Graph shows the average number of protrusions per cell relative to control ***p ≤ 0.001. **f** T47D cells were transfected with the indicated siRNAs. After 72 h, cells were fixed and stained for F-actin (Alexa Fluor 546 phalloidin) and DNA (DAPI). Cell area was measured using ImageJ from F-actin-stained images. Scatter dot plots show mean +/− SEM; *n* = 3, **p ≤ 0.01, ***p ≤ 0.001, Student’s *t* test. Relative RhoH mRNA levels in T47D cells were determined by qPCR using GAPDH as control. Mean +/− SEM, *n* = 3, ***p ≤ 0.001, Student’s *t* test. **g** RhoH depletion leads to an increase in binucleated cells. Cells were fixed 72 h after siRNA transfection and stained for F-actin (Alexa Fluor 546 phalloidin) and DNA (DAPI). The % of cells with two or more nuclei were scored; at least 250 cells per condition were analysed from five independent experiments. Graphs show mean +/− SEM, *n* = 5, *p ≤ 0.05, ***p ≤ 0.001, Student’s *t* test. **h** RhoH depletion reduces cell number. PC3 cell numbers were counted 96 h after siRNA transfection. Graphs show mean +/− SEM, *n* = 3, *p ≤ 0.05, **p ≤ 0.01, Student’s *t* test

**Fig. 7:**
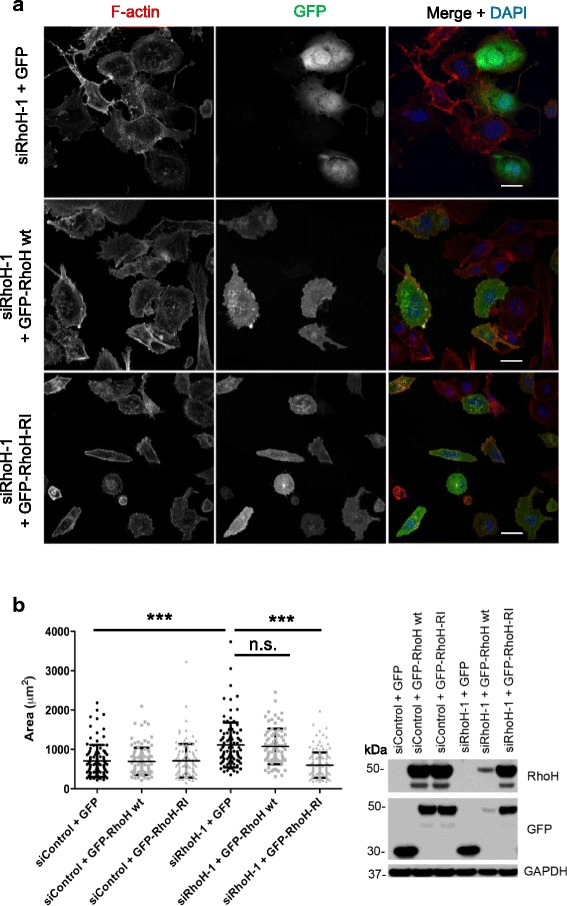
GFP-RhoH rescues the morphological phenotype of RhoH-depleted cells. **a** Representative images of PC3 cells transfected with GFP, GFP-RhoH-wt or GFP-RhoH-R1 (resistant to siRhoH-1) 48 h after siRNA transfection with siRhoH-1 or control siRNA. Cells were fixed 24 h after plasmid transfection and stained for F-actin with Alexa Fluor 546 phalloidin and for DNA with DAPI. Scale bars 20 μm. **b** *Left*, cell area was measured using ImageJ from F-actin-stained images. Scatter dot plots show mean +/− SEM; at least 84 cells/condition were analysed from three independent experiments, *n.s.* not significant, ***p ≤ 0.001, by one-way analysis of variance (ANOVA). *Right*, representative immunoblot from cell lysates, probed with the indicated antibodies. GAPDH was used as a loading control

To investigate how RhoH affects cell migration, we analysed the effect of RhoH knockdown on cell shape and polarity. RhoH depletion with both siRNAs increased cell spread area and reduced elongation in PC3 cells (Fig. 6a–d), both of which correlate with a loss of migratory polarity [30]. It also increased the number of membrane protrusions around the cell periphery (Fig. 6e), consistent with reduced migratory polarity. Similar to PC3 cells, RhoH depletion increased spread area in two other cell lines that express RhoH: T47D breast cancer cells (Fig. 6f) and LNCaP prostate cancer cells (unpublished data). Expression of siRhoH-1-resistant GFP-RhoH (GFP-RhoH-R1) rescued the increased spread area of siRhoH-1-transfected cells (Fig. 7a, b), indicating that the effect is specifically due to RhoH depletion. We conclude that RhoH-depleted cells have a reduced ability to assume a migratory polarity and hence have decreased migration speed.

Interestingly, RhoH depletion increased the proportion of binucleated cells in PC3 cells (Fig. 6a, g) as well as in T47D and LNCaP cells (unpublished data), indicating that RhoH depletion might inhibit cytokinesis [32]. This could be a consequence of the increased spread area of RhoH-depleted cells, which could inhibit cell rounding during cytokinesis. For example, compounds such as Rho kinase (ROCK) inhibitors both increase spread area and inhibit cytokinesis [32, 33]. Consistent with an inhibition of cytokinesis, the total cell number was lower 96 h after RhoH siRNA transfection compared to control cells, but not at 48 or 72 h (Fig. 6h).

### RhoH alters Rac activity distribution and membrane protrusion

**Fig. 8:**
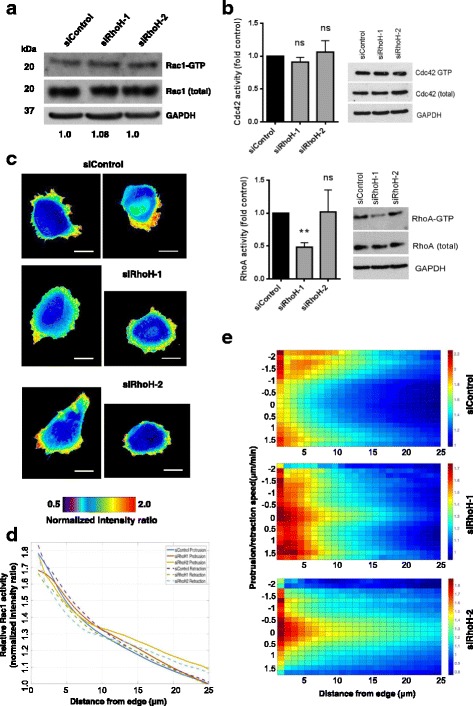
RhoH alters the distribution of active Rac1. **a** RhoH does not affect total Rac1 activity. PC3 cells transfected with the indicated siRNAs were analysed for Rac1 activity using GST-PBD and pulldown assays. Blot is representative of three independent experiments. The ratio of GTP-Rac1/Total Rac1 (compared to siControl) is indicated under the blot panels. **b** RhoH does not affect RhoA or Cdc42 activity. PC3 cells transfected with the indicated siRNAs were analysed for Cdc42 (*top*) or RhoA (*bottom*) activity using GST-RBD or GST-PBD beads and pulldown assays. Blot is representative of three independent experiments. Graph shows the ratio of GTP-RhoA/Total RhoA or GTP-Cdc42/Total Cdc42 (compared to siControl); *n* = 3, *ns* non-significant, **p ≤ 0.01, Student’s *t* test. **c**–**e** RhoH affects Rac1 activity distribution. 24 h after transfection with the indicated siRNAs, cells were transfected with DNA encoding a Rac1 biosensor including YPet PAK1-PBD and CyPet Rac1. Cells were imaged 24 h later by time-lapse microscopy on a wide-field microscope by epifluorescence. **c** Two example images of Rac1 activity distribution are shown for each siRNA. Scale bars 20 μm. **d** Graph of line scans (12 cells × 60 frames per cell) showing Rac1 activity with respect to distance from the edge of the cell and also depending on whether the cell edge was protruding or retracting. **e** Rac1 activity as a function of distance from cell edge and speed of membrane protrusion or retraction, *n* = 12 cells per condition


Since increased cell spread area and loss of cell polarity are induced by active Rac1 in PC3 cells [20, 30], we investigated whether RhoH affected Rac1 activity. Indeed, RhoH has previously been reported to regulate Rac activity in haematopoietic progenitor cells (HPCs) [34]. However, RhoH depletion did not affect total Rac1 activity in PC3 cells, nor did it alter total Cdc42 or RhoA activity (Fig. 8a, b). To investigate whether RhoH affected where Rac1 is active in cells, we used a fluorescence resonance energy transfer (FRET)-based Rac1 activity FLARE biosensor [35, 36]. In control siRNA-transfected cells, Rac1 activity was generally polarised and localised to one or two discrete areas of the cell periphery. In contrast, in RhoH-depleted cells, Rac1 activity was more uniformly localised around the cell periphery (Fig. 8c; Additional file 5: Movie S4, Additional file 6: Movie S5 and Additional file 7: Movie S6).

**Additional file 5: Movie S4.** Rac1 biosensor in PC3 cells transfected with siControl. One frame every 10 s. Scale bar 10 μm. (MP4 1231 kb)



**Additional file 6: Movie S5.** Rac1 biosensor in PC3 cells transfected with siRhoH-1. One frame every 10 s. Scale bar 10 μm. (MP4 1645 kb)



**Additional file 7: Movie S6.** Rac1 biosensor in PC3 cells transfected with siRhoH-2. One frame every 10 s. Scale bar 10 μm. (MP4 1631 kb)


Automated line scan analysis was carried out to quantify Rac1 biosensor activity at the edge of cells [37]. Rac1 activity was highest close to the cell edge in PC3 cells, as previously reported in mouse embryonic fibroblasts [35] (Fig. 8d). Rac1 activity was correlated with protrusion and retraction rate of the plasma membrane. In control PC3 cells, Rac1 was active in protruding and retracting regions of cells (Fig. 8d, e). By contrast, in RhoH-depleted cells, Rac1 was also active in regions of the membrane that were neither protruding nor retracting (Fig. 8e). These results indicate that RhoH functions to enhance Rac1 coupling to membrane protrusion, explaining why RhoH-depleted cells are impaired in cell migration. Although Rac1 is active in these cells, its activity does not correlate with membrane protrusion.

### RhoH co-localises with and associates with Rac1 and PAK2

**Fig. 9:**
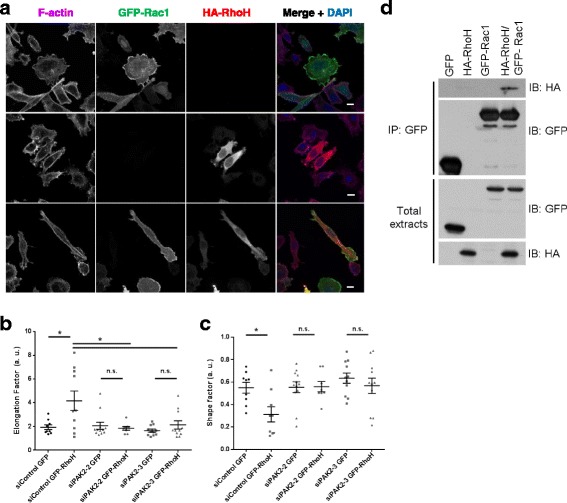
RhoH co-localizes and interacts with Rac1. **a** RhoH partially co-localizes with Rac1. Cells were seeded on Matrigel-coated coverslips and transfected with GFP-Rac1 and/or HA-RhoH as indicated. After 24 h cells were fixed and stained for F-actin with Alexa Fluor 633 phalloidin, for anti-HA antibodies for HA-RhoH and for DNA with DAPI. Images were acquired by confocal microscopy. Scale bar 10 μm. **b**, **c** RhoH promotes cell elongation, which is not affected by PAK2. 48 h after siRNA transfection with siPAK2-2/3 or control siRNA, cells were transfected with GFP or GFP-RhoH. Cells were fixed and stained for F-actin with Alexa Fluor 546 phalloidin and DNA with DAPI. Cell elongation factor and shape factor were measured using ImageJ from F-actin-stained images. Scatter dot plots show mean +/− SEM; cells were analysed from three independent experiments, *n.s.* non-significant, *p ≤ 0.05, Student’s *t* test. **d** Rac1 and RhoH co-immunoprecipitate. COS7 cells were transfected with constructs encoding GFP, HA-RhoH and/or GFP-Rac1 as indicated. After 24 h, lysates were immunoprecipitated with anti-GFP and analysed by western blotting with the indicated antibodies

Given that RhoH alters the localisation of active Rac1, we investigated whether RhoH co-localises with Rac1. We observed that RhoH localised in discrete dots in the cytoplasm (which could be membrane vesicles) and at the edges of lamellipodia (Fig. 9a). RhoH expression promoted cell elongation and cell polarity (Fig. 9b, c), consistent with a role in regulating Rac1 activity. When co-expressed with Rac1, RhoH co-localised with Rac1 in lamellipodia and membrane ruffles, as well as to vesicular structures (Fig. 9a). RhoH also co-immunoprecipitated with Rac1, indicating that they associate in cells (Fig. 9d).

**Fig. 10:**
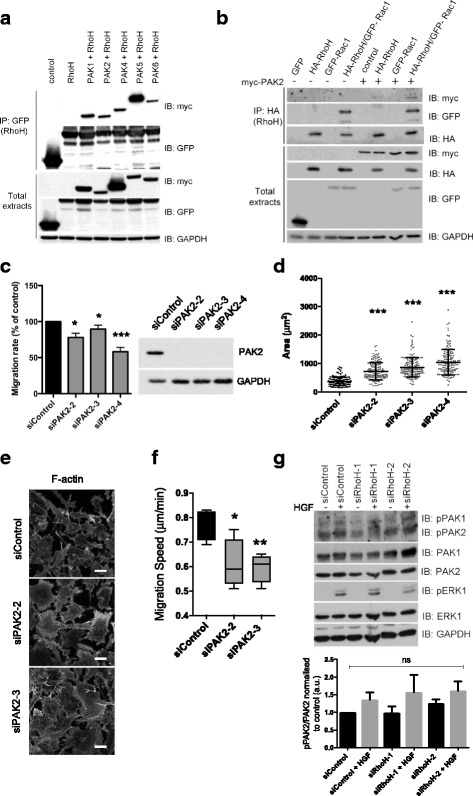
PAK2 mediates the effects of RhoH on cell shape. **a**, **b** RhoH co-immunoprecipitates with PAKs. COS7 cells were transfected with constructs encoding GFP-RhoH and myc epitope-tagged PAKs (**a**) or HA-RhoH, GFP-Rac1, Myc-PAK2 or GFP alone (**b**). After 24 h, lysates were incubated with GFP-Trap beads (GFP IP) followed by western blotting with the indicated antibodies. Results shown are representative of three independent experiments. **c** PAK2 depletion reduces cell migration. PC3 cells were transfected with the indicated siRNAs and Oris™ migration assay carried out as in Fig. 2. Migration is shown as a percentage of siControl +/− SEM, *n* = 3; *p ≤ 0.5, ***p ≤ 0.001, Student’s *t* test. Representative immunoblot from cell lysates probed with the indicated antibodies. GAPDH was used as loading control. **d**–**f** PAK2 depletion increases cell area and decreases migration. PC3 cells were transfected with the indicated siRNAs. **d** After 72 h, cells were fixed and stained for F-actin (Alexa Fluor 546 phalloidin) and DNA (DAPI). Cell area was measured from F-actin-stained images. Scatter dot plots show mean +/− SEM; *n* = 3 (> 125 cells/condition), ***p ≤ 0.001, Student’s *t* test. **e** Representative images used for **d**; scale bar 10 μm. **f** Cells were seeded 48 h after transfection in RPMI containing 0.1% FCS and monitored by time-lapse microscopy for 24 h, images taken every 5 min. At least 50 cells were tracked from two independent experiments. *Boxes* of box and whisker plots show median, 25th and 75th percentiles; *whiskers* show minimum and maximum values; *p ≤ 0.05, **p ≤ 0.01, Student’s *t* test. **g** RhoH does not alter PAK activity. PC3 cells were transfected with the indicated siRNAs, cultured for 24 h in medium containing 0.1% FCS then stimulated with 20 ng/ml HGF for 10 min. Cell lysates were probed with the indicated antibodies. Anti-phospho-PAK recognises PAK1, PAK2 and PAK3. GAPDH was used as loading control. Graph shows densitometric quantification of western blots. *n* = 3; *ns* non-significant, Student’s *t* test

Of the Rho GTPase effectors that were hits in our siRNA screen (Table 1), PAK2 and PAK5 (named PAK7 in the screen) are potential RhoH as well as Rac1 interactors. PAK1 and PAK5 have previously been reported to interact with RhoH [38, 39]. All of the PAK proteins we tested (PAK1, PAK2, PAK4, PAK5 and PAK6) co-immunoprecipitated with GFP-RhoH (Fig. 10a), but GFP alone did not pull down either PAK1 or PAK2 (data not shown), indicating that the interaction is specific. PAK2 is more highly expressed than PAK1 in PC3 cells [40], which may explain why PAK2 but not PAK1 was a hit in our screen. Both PAK2 and Rac1 were detected in RhoH immunoprecipitates (Fig. 10b), indicating that Rac1, PAK2 and RhoH might form a ternary complex.

Like RhoH depletion, PAK2 depletion reduced migration in the modified scratch wound assay (Fig. 10c) and increased cell spread area (Fig. 10d, e). Our previous studies showed that PAK2 depletion reduced HGF-induced PC3 cell migration [40]. PAK2 depletion with two independent siRNAs also reduced migration speed and distance migrated in the absence of HGF stimulation (Fig. 10f). PAK2 depletion reduced GFP-RhoH-induced PC3 cell elongation, indicating that PAK2 mediates morphological responses to RhoH (Fig. 9b, c). However, RhoH did not affect the level of phosphorylated PAK, using an antibody that is a readout for PAK activity, because it detects auto-phosphorylated PAK1 (residues 199/204), PAK2 (residues 192/197) and probably PAK3 (Fig. 10g).

**Fig. 11:**
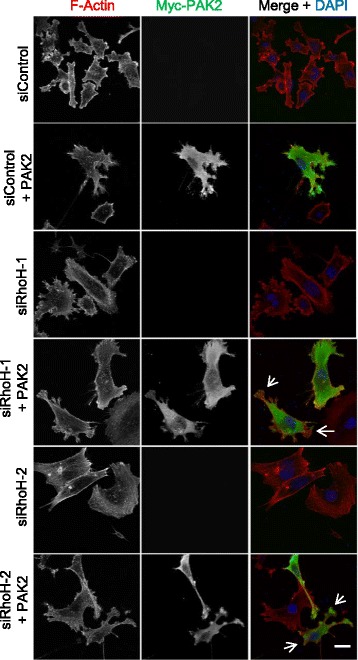
RhoH depletion alters PAK2 localisation and PAK2-induced lamellipodia. PC3 cells were transfected with the indicated siRNAs. After 48 h, cells were transfected with empty vector or vector encoding myc-PAK2. Cells were fixed 24 h later and stained for F-actin with Alexa Fluor 546 phalloidin, anti-myc antibodies for Myc-PAK2 and DNA (DAPI). Representative images of cells are shown. Scale bar 10 μm. *Arrows* indicate protrusions with no PAK2 or in which PAK2 does not overlap with F-actin

We then investigated whether RhoH altered PAK2 localisation (Fig. 11). PAK2 overexpression in PC3 cells induced multiple lamellipodial protrusions, and PAK2 localisation was enriched in these protrusions (as previously described for PAK1 in Swiss 3T3 cells [41]). RhoH depletion altered PAK2 localisation and reduced PAK2-induced lamellipodia (Fig. 11, see arrows).

Together our data indicate that RhoH associates with both Rac1 and PAK2 to promote cell migratory polarity.

## Discussion

The RNAi screen we report here has identified multiple regulators of cell migration in prostate cancer cells, including Rho GTPases, RhoGEFs, RhoGAPs and effectors. siRNA pools targeting closely related RhoGEFs, RhoGAPs and effectors give opposite phenotypes, indicating that they are not functionally redundant but have distinct roles in cell signalling. Unexpectedly, RhoH was a hit in the screen that reduced cell migration when it was depleted. Our observations show that RhoH-depleted cells lose migratory polarity and that this correlates with de-localised Rac1 activation around the cell periphery. We show that RhoH associates with both Rac1 and the Rac1 target PAK2, and co-localises with Rac1 in lamellipodia. In addition, PAK2 contributes to the effects of RhoH on cell shape and migratory polarity, indicating that RhoH acts together with Rac1 and PAK2 to stimulate cell migration.

Previous expression analysis implied that RhoH is expressed only in haematopoietic cells, and thus it was surprising that it had a phenotype in a prostate cancer cell line. RhoH function has been investigated primarily in T cells and HPCs [19]. Whereas RhoH is required for optimal migration of PC3 cells, it appears to suppress migration of HPCs. For example, mouse HPCs from *rhoH*-null mice have increased migration and chemotaxis, which correlates with increased Rac1 activity [33, 42]. The contribution of RhoH to T cell migration appears to depend on both the concentration of chemokine and the presence of T cell receptor signalling, which also involves RhoH [11, 38, 43, 44] and Rac [19]. RhoH overexpression inhibits chemotaxis of T cells, and RhoH depletion increases their migration speed in vitro and homing to lymph nodes in vivo [43]. However, at high levels of the chemokine CXCL12, Jurkat T cell migration depends on RhoH, whereas at low levels of CXCL12 it suppresses cell migration [38]. Thus, under certain conditions, RhoH is a positive regulator of T cell migration, similar to our observations in prostate cancer cells. It is also possible that constitutive loss of RhoH in cells derived from *rhoH*-null mice selects for cells with higher Rac1 activity, to compensate for the effects of RhoH loss in reducing Rac1-induced protrusion. By contrast, under our conditions of acute RhoH depletion, the cells do not have time to adjust their signalling networks to Rac1, and hence have reduced migration speed.

RhoH has previously been reported to interact with PAK1 and PAK5 [38, 39], but the phenotypic consequence of this interaction has not been determined. Here, we show that RhoH associates with both Rac1 and PAK2, and acts through PAK2 to regulate migratory polarity in PC3 cells. Although RhoH affects the distribution of active Rac1 and co-localises with Rac1 in lamellipodia, Rac1 itself is not a hit in our migration assay (Table 1), possibly because Rac2 is also expressed in PC3 cells. Indeed, Rac2 can induce lamellipodia similar to Rac1 [45]. Surprisingly, Rac3 depletion increased migration. Rac1 and Rac3 have previously been reported to have opposite effects on neuronal cell adhesion [46], and only Rac1 and not Rac3 depletion reduced lamellipodia in glioblastoma cells [47]. It is thus probable that Rac3 acts in a different way than Rac1 and Rac2 in regulating cell migration.

Our analysis of prostate cancer gene expression data suggests that higher RhoH expression correlates with more rapid cancer progression, and thus the role of RhoH in prostate cancer warrants further investigation. Interestingly, PAK2 was found to be more active in castration-resistant prostate cancers compared to castration-sensitive cancers and to contribute to prostate cancer cell invasion as well as proliferation in vitro [48]. This implies that PAK2 could act with RhoH to promote cancer progression.

Similar to RhoH depletion, knockdown of either RhoC or the ezrin-radixin-moesin (ERM) protein radixin in PC3 cells leads to an increase in spread area [20, 30]. In both RhoH- and RhoC-depleted cells, Rac1 is uniformly activated around the periphery rather than in discrete areas in control cells. RhoC depletion also prevents cell polarisation and strongly inhibits random migration [30] as well as in the modified scratch wound assay (our data; Table 1). However, RhoH-depleted cells have small and persistent areas of active membrane protrusion and retraction around the periphery, whereas RhoC-depleted cells tend to ruffle all around the periphery. Thus, they are likely to affect cell spread area by different mechanisms. In radixin-depleted PC3 cells, global Rac1 activity is increased via Vav GEFs [20], whereas RhoH or RhoC depletion does not affect global Rac1 activity.

During cell attachment to an extracellular matrix, cells initially spread uniformly and then start to polarise and migrate. During this initial phase, Rac1 is active throughout the cell periphery, inducing Arp2/3 complex-mediated lamellipodial protrusion to drive spreading [49]. We propose that RhoH-depleted cells rarely switch from this uniform spreading mode to a polarised migratory morphology, because they have a reduced ability to turn off Rac1 around the periphery so that it is localised to a specific area in order to form a leading edge, which in turn involves PAKs. These highly spread cells represent the population of RhoH-depleted cells that migrate very little. Rac1 remains uniformly active on the plasma membrane, and since the cells have already spread, this active Rac1 is no longer able to drive membrane protrusion at all places where it is active. Instead, it would stimulate protrusion only in focal regions around the periphery where the membrane has stochastically retracted.

Our work implies that acquisition of RhoH expression in epithelial cancers would promote cell migration and invasion by restricting Rac1 activity and hence protrusion to one area of the plasma membrane. This would increase the persistence of cancer cell migration through tissues and ultimately contribute to cancer progression and metastasis.

## Methods

### Cell culture and transfection

PC3, LNCaP, T47D and DU145 cells were cultured in RPMI-1640 medium containing 10% FCS. MDA-MB-231, MCF7, BT20, HT29, A376M2 and COS7 cells were cultured in Dulbecco’s modified Eagle’s medium (DMEM) containing 10% FCS. MCF10A cells were cultured in DMEM/F12 medium supplemented with 5% horse serum, 20 μg/ml epidermal growth factor (EGF), 100 ng/ml cholera toxin, 100 μg/ml insulin and 0.5 μg/ml hydrocortisone. SUM159 cells were cultured in F12 medium supplemented with 5% FCS, 5 μg/ml insulin and 1 μg/ml hydrocortisone. All media were supplemented with 100 U/ml penicillin and 50 μg/ml streptomycin.

PC3 cells were transfected with siRNAs using Oligofectamine (Invitrogen) in Optimem without antibiotics. The medium was changed to RPMI-1640 growth medium with antibiotics 6 h after transfection. siRNAs were purchased from Dharmacon or Sigma-Aldrich. Sequences of siRNAs are shown in Additional file 1: Table S1. Plasmids were transiently transfected into PC3 cells using Lipofectamine 2000 (Invitrogen).

### Plasmid construction and mutagenesis

The human astrocyte (HA)-epitope-tagged RhoH gene in pRC/CMV was a kind gift from Jeffrey Settleman (Massachusetts General Hospital, Boston, MA, USA). HA-RhoH was mutated to make it resistant to siRhoH-1 (GFP-RhoH-R1) by site-directed mutagenesis, using a QuikChange mutagenesis kit (Stratagene). The nucleotide change was verified by DNA sequencing (Eurofins-MWG, UK). Wild-type RhoH and RhoH-R1 were subcloned into pEGFP-N.

### RNA isolation, PCR and qPCR

RNA was isolated from cells using RNeasy Mini Kits (Qiagen). Contaminating DNA was used with a DNase-free kit (Ambion). For complementary DNA (cDNA) synthesis, a SuperScript VILO Kit was used (Invitrogen). qPCR was carried out with cDNA using SYBR Green-containing Master Mix (Primer Design). *GADPH* was used as a reference gene. The qPCR oligonucleotide primers used for *RhoH* were as follows: F, GAGAAGTAACATTCTGCAAATCGC R, AGCACACGCCATTCAGCAAG; for *Rac2*: F, GCAAGACCTGCCTTCTCATCA R, GCTGTCCACCATCACATTGG; for *RhoA*: F, CAACTATGATTATTAACGATGTCCAACC R, TGGTGTGTCAGGTGGGAGTG; and for *GAPDH*: F, GTGAAGGTCGGAGTCAACG R, TGAGGTCAATGAAGGGGTC.

### Migration assay and screen conditions

PC3 cells were incubated with 2 μM CFSE (Molecular Probes) for 20 min in phosphate-buffered saline (PBS) followed by incubation in medium for 15 min, then washed to remove excess dye. Cells (8 × 10^4^/well) were transfected in suspension, in 96-well dishes containing 50 nM siRNA pools or 100 nM single siRNAs, with Lipofectamine 2000 in 150 μl of Optimem for 16 h. After siRNA transfection, 3 × 10^4^ cells from each transfection well were seeded around Oris inserts in each of two wells on 96-well plates, in medium containing 1% FCS. Where indicated, wells were pre-coated with Matrigel (100 μg/ml; BD Biosciences). The inserts were removed 48 h after siRNA transfection, and fluorescence images were acquired for each well (*t* = 0 h), using a TE2000 Nikon inverted microscope with a motorised stage (Prior) and 4× objective. HGF (20 ng/ml, PeproTech, Rocky Hill, NJ, USA) was added to one of the two wells for each siRNA transfection. After 24 h, a second fluorescence image was acquired from the same position. The fluorescence images were thresholded to select only the cells, and the migration area was determined as the area free of cells at 0 h. The migration index was calculated as the percentage of the migration area covered with cells (fluorescent pixels) 24 h after removing the stoppers. The mean and standard deviation (SD) of three independent experiments were calculated for each siRNA. A Z-score was allocated to each siRNA, where the Z-score is the difference between the mean migration index for an siRNA and the mean for all the siRNAs in the plate divided by the SD of all the siRNAs in the plate. A Z-score of 1.0 was selected to classify the different siRNAs into three categories: impaired migration (those siRNAs with Z-score < −1.0), accelerated migration (those siRNAs with Z-score > 1.0) or HGF-dependent (if the Z-score was > −1.0 in normal medium but < −1.0 in medium supplemented with HGF).

To test for the effects of siRNAs on cell viability, CFSE-stained cells (2 × 10^4^) transfected with siRNA pools were seeded per well in 96-well plates. Fluorescence was measured on a Fusion α-FP Plate Reader (Perkin Elmer) at 48 and 72 h after transfection. A viability index corresponding to the 72 h/48 h fluorescence ratio was calculated for each siRNA. Z-scores for the viability of the cells were allocated to each siRNA, where the Z-score is the difference between the mean viability index for an siRNA and the mean for all the siRNAs in the plate divided by the SD of all the siRNAs in the plate. A Z-score of 1.0 was selected to classify the different siRNAs into low viability (Z-score < −1.0) and good viability (Z-score > 1.0).

### Time-lapse microscopy and random migration analysis

For random migration, PC3 cells were seeded on a 6-well or a 24-well plate 24 h after siRNA transfection, and 24 h later images were acquired at 37 °C every 8 min for 24 h (Fig. 5a, b) or every 4 min for 24 h (Fig. 5c) on a Nikon TE2000 microscope with a Prior stage and a Hamamatsu ORCA camera controlled by MetaMorph software (Molecular Devices, San Jose, CA, USA), using a 10×/0.3 Plan Fluor objective. Cells were imaged in medium containing 1% FCS (Fig. 5a, b) or in 0.1% FCS with or without 20 ng/ml HGF (Fig. 5c). More than 50 cells per siRNA condition (Fig. 5a, b) or 100 cells per condition (Fig. 5c) from three different experiments were tracked using ImageJ software. Still images are from 72 h after transfection.

The cell tracks were analysed by pooling all the data from all cells for each condition (siControl, siRhoH-1, siRhoH-2; Fig. 5a, b). From these data the distribution of the distance travelled in the *x*-direction between image frames was calculated (Fig. 5d). These numbers were compared to predictions from a mathematical model of cell migration. This model is a random walk in two dimensions, with memory of orientation from the prior step. The model is defined by the following parameters: mean speed, variance in speed and variance in cell orientation. Initially the model could not satisfactorily capture the data. We found that hypothesizing the existence of two ‘states’ of migration improved the model fit such that data were adequately described. Each migration state has three parameters: mean speed, variance in speed and variance in orientation. A fourth parameter, α, is the fraction of time spent (mean across all cells) in the first state. By fitting the model to the data, estimates for each of these parameters were obtained.

### Transwell migration assays

PC3 cells (8 × 10^4^) were seeded 48 h after siRNA transfection per transwell (8-μm pore diameter, BD Biosciences) in 0.1% FCS. The bottom well contained 1% FCS, a chemoattractant. After 8 h, cells were fixed with 70% ethanol containing 0.2% crystal violet. Random images of cells on the bottom of the transwell (10 per experimental condition from each of three independent experiments) were acquired using a Nikon Eclipse TS100 microscope with a 10× objective. Cells were counted using ImageJ (plugin Cell Counter). For each experimental condition, the total number of cells from the 10 images was divided by the total number of cells for control siRNA-transfected cells.

### Cell lysis, immunoprecipitation and western blotting

Cells were lysed 72 h after siRNA transfection in lysis buffer A (50 mM Tris-HCl pH 8, 0.5 mM ethylenediaminetetraacetic acid (EDTA), 150 mM NaCl, 1% NP-40 and protease and phosphatase inhibitor cocktails from Roche). Lysates were homogenised through a needle or sonicated, then centrifuged for 10 min at 16,000 g to separate soluble from insoluble fractions.

For immunoprecipitations, cells were lysed 24 h after plasmid transfection in lysis buffer B (20 mM Tris-HCl, pH 8, 130 mM NaCl, 1% Triton X-100, 1 mM dithiothreitol (DTT), 10 mM NaF, 1 mM phenylmethylsulfonyl fluoride, 10 μg/ml aprotinin, 10 μg/ml leupeptin, 0.1 mM sodium orthovanadate), and the centrifuged supernatants were incubated with GFP-Trap beads (ChromaTek) or mouse anti-HA antibody bound to agarose (Sigma-Aldrich) and rotated for 2 h at 4 °C. The beads were washed extensively with lysis buffer, and the bound proteins were analysed by sodium dodecyl sulfate polyacrylamide gel electrophoresis (SDS-PAGE) and immunoblotting. Bound antibodies were visualised with horseradish peroxidase-conjugated goat anti-immunoglobulin G antibodies and enhanced chemiluminescence (ECL, GE Healthcare).

The antibodies used for western blotting were GAPDH (RRID:AB_2107445), phospho-PAK1/2 (S199/204 on PAK1; S192/197 on PAK2; RRID:AB_2160222), PAK1 (RRID:AB_10827914), PAK2 (RRID:AB_228338), RhoH (RRID: AB_1856269), GFP (RRID:AB_221569), Cdc42 (RRID:AB_2078085), Rac1 (RRID:AB_309712), RhoA (RRID:AB_10693922), α-tubulin (RRID:AB_477593), HA (RRID:AB_10094468 RRID:AB_2314672), myc epitope (RRID:AB_439680), ERK1 (RRID:AB_2140110) or phospho ERK1/2 (RRID:AB_331646). HRP-labelled goat anti-mouse, anti-rat and anti-rabbit antibodies were also used (GE Healthcare).

### GTPase activity assays

GST-PBD (p21-binding domain of PAK) for Rac1 and Cdc42 activity assays or GST-RBD (Rho-binding domain of Rhotekin) for RhoA activity assays was produced by lysing bacterial pellets in cold STE buffer (10 mM Tris pH 8.0, 150 mM NaCl, 1 mM EDTA, 1 mM phenylmethylsulfonyl fluoride). Lysates were homogenised using a 19-gauge needle, then lysozyme (100 μg/ml) was added and incubated for 15 min on ice. DTT (5 mM) was added followed by Tween-20 (1%) and SDS (0.03%). The homogenate was centrifuged at 13,000 g at 4 °C for 30 min. The supernatant was transferred to a fresh tube containing glutathione-conjugated sepharose beads and rotated for 2 h at 4 °C. The beads were washed in STE buffer followed by Mg^2+^ lysis buffer (25 mM Hepes, pH 7.5, 150 mM NaCl, 1% NP-40, 10 mM MgCl_2,_ 1 mM EDTA, 25 mM NaF, 1 mM Na_3_VO_4,_ 1 mM phenylmethylsulfonyl fluoride, 10% glycerol and two cocktail protease inhibitor tablets (Roche)).

PC3 cells were transfected with 100 nM siRhoH-1, siRhoH-2 or control siRNA. After 72 h, the cells were washed twice with ice-cold PBS and lysed in ice-cold Mg^2+^ lysis buffer. Cell lysates were centrifuged for 5 min at 13,000 g at 4 °C, and 40 μl of supernatant was removed to determine total Rac1, Cdc42 or RhoA levels. The remaining supernatants were incubated with GST-PBD or GST-RBD on glutathione-sepharose beads and rotated at 4 °C for 2 h. The beads were washed extensively in lysis buffer, and the bound proteins were separated by SDS-PAGE and immunoblotted with anti-Rac1 (RRID:AB_309712), anti-Cdc42 (RRID:AB_2078085) or anti-RhoA (RRID:AB_10693922) antibodies respectively. Blots were quantified using ImageJ.

### Cell staining, confocal microscopy and cell shape analysis

PC3 cells on Matrigel-coated glass coverslips were fixed with 4% paraformaldehyde solution in PBS for 20 min, permeabilised with 0.1% Triton X-100 in PBS for 5 min and blocked with 5% bovine serum albumin (BSA) in PBS for a further 30 min. Cells were incubated for 1 h with Alexa Fluor 546-conjugated phalloidin (Molecular Probes) to stain actin filaments (F-actin) and fluorescein isothiocyanate (FITC)-labelled mouse α-tubulin antibody (RRID:AB_476967), anti-HA antibody (RRID:AB_10094468 or RRID:AB_2314672), anti-myc epitope antibody (RRID:AB_439680) or anti-GFP antibody (RRID:AB_221569) and 4′,6-diamidino-2-phenylindole (DAPI) to stain nuclei. When necessary, bound antibodies were visualised with anti-rabbit and anti-rat Alexa Fluor 546/488 conjugated antibodies. Coverslips were mounted on slides in fluorescence mounting medium (Dako), and images were generated with a Zeiss LSM Zen 510 confocal microscope using a 40×/1.3 NA objective and Zen software.

For cell shape analysis, cells stained for F-actin with phalloidin were analysed. ImageJ was used to calculate cell spread area, elongation and roundness (shape factor). Shape descriptors were calculated using ImageJ or MetaMorph software and are defined as follows: Circularity = [Area]/[Perimeter], with a value of 1.0 indicating a perfect circle; as the value approaches 0.0, it indicates an increasingly elongated shape. Shape factor = 4πA/P2; a value near 0 indicates a flattened object, whereas a value of 1.0 indicates a perfect circle. Elongation factor = 1/Shape factor.

### Cell number analysis

PC3 cells (6–8 × 10^4^) were plated on 6-well tissue culture dishes that were pre-coated for 1 h with 100 μg/ml Matrigel (BD Biosciences). After 16 to 24 h, the cells were transfected with 100 nM siRNAs. Cells were counted at 48 h, 72 h or 96 h after transfection using a haemocytometer.

### Rac1 activity analysis by FRET

PC3 cells plated on Matrigel-coated coverslips were transfected with 100 nM siRNAs, followed by transfection with a FLARE biosensor for Rac1, 48 and 24 h before imaging respectively. The FLARE biosensor was a modification of that previously described [35]. It consisted of a PAK1 PBD domain fused to YPet followed by CyPet-Rac1, on one open reading frame. A 2(P)A ‘self-cleaving’ sequence from FMDV was inserted, leading to cleavage of PBD-YPet from CyPet-Rac1 during translation [50]. For imaging, phenol red-free RPMI-1640 medium supplemented with 1% FCS was used. Cells were imaged in a chamber heated to 37 °C using an Olympus IX81 inverted microscope with a 40×/1.3 NA objective fitted with an objective heater (Bioptechs). Cyan fluorescent protein (CFP) and FRET images were acquired simultaneously every 10 s for 10 min through two CoolSNAP HQ2 CCD cameras (Photometrics) using a TuCam camera adapter (Andor Technology). Image processing and FRET analysis were carried out as previously described [51].

### Line scan analysis and correlation with cell edge protrusion/retraction speed analysis

Line scan analysis was performed on FRET images of 12 cells from three independent experiments. FRET intensity of line scans over 60 frames per cell acquired every 10 s was analysed as described [37] and plotted as a function of distance from the cell edge. The correlation between the protrusion and retraction speed at the cell edge and the FRET signal was also calculated [37].

### Analysis of RhoH expression in prostate cancers

The MSKCC prostate cancer mRNA expression dataset and descriptive statistics of associated clinical data were obtained from the cBio Cancer Genomics Portal [52]. RhoH mRNA expression of samples were divided into terciles, with the top tercile designated as high-RhoH and the rest of the dataset as low-RhoH. The data were fitted to a Cox proportional hazards regression model to estimate RFS with RFS defined as time until PSA levels increased to ≥ 0.2 ng/ml [31]. The Wald test was used to assess significance. Statistical analyses were performed using the statistical environment R (http://www.r-project.org/).

### Statistical analysis

All data are presented as mean plus SEM. The statistical analysis was performed using GraphPad Prism software. The statistical significance analysis was calculated using an unpaired two-tailed *t* test (for comparing two conditions) or by one-way analysis of variance (ANOVA) with multiple conditions, or a Wald test (for survival analysis).

## Conclusions

Using an RNAi screen, we show that multiple Rho GTPases and their interacting partners, including GEFs, GAPs and effectors, regulate cell migration in PC3 prostate cancer cells. One of the screen hits is RhoH, which was assumed to be expressed only in haematopoietic cells, but we show is expressed in a panel of epithelial cancer cell lines. RhoH promotes cell migration and chemotaxis of PC3 cells. RhoH co-localises with and associates with Rac1 and PAK2, a Rac1 effector and a hit in our RNAi screen, and promotes coupling of Rac1 activity to membrane protrusion events as well as PAK2 localisation to lamellipodia. We propose that RhoH stimulates Rac1- and PAK2-driven membrane protrusion and hence enhances cell migration.

## Additional files

Additional file 1: **Table S1**. List of gene targets in siRNA library. Genes are divided into controls, Rho GTPases, other GTPases, RhoGEFs, RhoGAPs, effectors and others. Gene names and alternative gene names, Entrez Gene GeneIDs, RefSeq accession numbers, siRNA sequences and effects of each gene on migration and cell viability are shown as Z-scores. (XLSX 49 kb)
